# Applying the Evidence Pyramid to Breastfeeding and Lactation Research

**DOI:** 10.1177/08903344251406587

**Published:** 2026-02-14

**Authors:** Melissa Ann Theurich, Elizabeth Kar

**Affiliations:** 1 Division of Neonatology, Dr. von Hauner Children’s Hospital, LMU University Hospital, Ludwig-Maximilians-Universität München, Germany; 2 North Dakota State University, Fargo, ND, USA

**Keywords:** breastfeeding, breastfeeding assessment, epidemiological study designs, evidence pyramid, methodology, public health, systematic reviews

## Introduction

This article emphasizes the role of the study design in effectively interpreting and applying breastfeeding and lactation research. Outside of scientific research, practitioners may rely on anecdotal evidence, which comes from their own practice, or word-of-mouth. While clinical experience is valuable, it is not considered scientific evidence. This is because clinical experiences are subjective, not recorded in a standardized manner and do not undergo quality checks, such as peer review. To judge the quality of scientific studies in breastfeeding and human lactation research, this article borrows from the long-established concept of an evidence pyramid (Shaneyfelt, 2016), which ranks studies by their “level of evidence,” based on their study designs. The objective of this article is to help lactation support practitioners critically assess the validity of research findings by putting available evidence into perspective based on their research design. By applying this hierarchical framework, practitioners can begin to navigate evolving, and often controversial, scientific evidence.

## Primary versus Secondary Research

There are two main types of research: primary and secondary research. In primary research, data is collected directly by the researchers. In secondary research, researchers analyze and synthesize data that have already been collected for routine monitoring purposes or that have been collected previously for another purpose. Secondary research summarizes the available evidence or characterizes the body of evidence from primary research to answer a specific research question.

One type of secondary research is evidence synthesis, which includes study designs like narrative reviews, scoping reviews, systematic reviews, or umbrella reviews. These types of secondary research have varying levels of rigor (e.g., systematic vs. non-systematic literature searches, assessments or no assessments of the quality of the primary studies summarized, etc.).

## Observational versus Experimental Study Designs

The study design determines the methods and the framework for how a scientific study is conducted. Some study designs are interventional studies, whereas others are observational. Using observational research designs, researchers simply observe and record what happens naturally without intervening or applying treatments or interventions (Thiese, 2014). For example, in an observational study design, researchers might examine how different hospital practices relate to breastfeeding success.

In experimental studies, researchers actively test something, like a new breastfeeding intervention (e.g., a new mobile app, or a novel nipple care regimen), to see how the invervention affects an outcome (e.g., breatfeeding duration, or wound healing). These types of studies are also called “experimental” studies because the researcher introduces a change and studies the result. In experimental study designs, practitioners may be aware of which treatment participants receive (unblinded), whereas other study designs may mask which participants receive a particular treatment to prevent any potential investigator bias (blinded). While blinding of the intervention is ideal (because it reduces the risk for investigator bias), it is not always possible for researchers and health providers to be blinded to the study groups, due to the nature of the intervention or other circumstances.

In breastfeeding and lactation research, observational studies are more common than intervention studies, primarly due to ethical considerations. For example, due to the well-established knowledge of the maternal and child health benefits of human milk feeding, a study would be unethical if it assigned some research participants to receive human milk, while others were assigned not to receive human milk. Study design is not only important for ethical and methodological considerations; it also helps readers to determine to what extent they can be confident in the validity of study results.

## The Evidence Pyramid for Breastfeeding and Human Lactation Research

**Figure 1.**
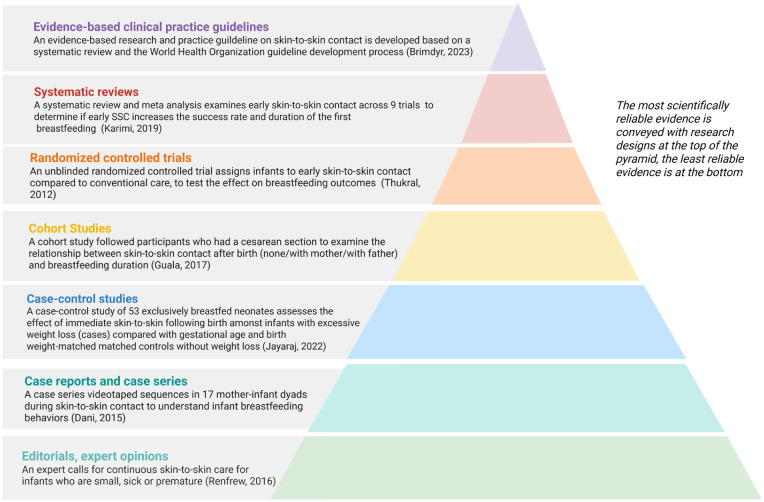
Evidence pyramid on the relationship between skin-to-skin care and breastfeeding using published research with various study designs.

An evidence pyramid is a visual tool used to depict a hierarchy of scientific evidence based on study design. We created an evidence pyramid using real-word examples of published research with various study designs to describe, examine, and experiment with the relationship between skin-to-skin contact and breastfeeding outcomes (see Figure 1).

Ranking breastfeeding and lactation research into a hierarchical scheme helps practitioners to put controversial and evolving scientific evidence into perspective. “Low-level evidence” is placed at the pyramid base, while “high-level evidence” is at the top of the pyramid. In the following sections, we describe common types of study designs. We reference oneexample of breastfeeding and lactation research for each type of study design and place it in the evidence pyramid (see Figure 1).

## Editorials, Expert Opinion

Editorials and expert opinions are ranked at the lowest level of the evidence pyramid. This is because these types of articles may be influenced by various types of bias. For example, scientific literature cited in an editorial is usually not chosen through systematic searches using standardized search methodologies or a variety of search databases. Therefore, an editorial may focus on one or a limited number of studies the author finds personally relevant.

Editorials and expert opinions may not give a balanced or comprehensive review of all the literature available on a given topic. They are usually written to outline an argument or rationale for a certain position, highlighting opinion and interpretation of available research and phenomena. They may explore perspectives that were not previously considered, make a case for a particular course of action or provide added support for a particular viewpoint. The ranking of this type of article at the base of the pyramid is not related to the quality of content but is only a reflection of the study design.

**Key Messages**

- Practitioners benefit from knowing the basics about research study designs, which puts research into context through determining the “level of evidence.”
- Regardless of the study design, studies should also be assessed based on their scientific rigor; there can be both good and poor studies at every level of the evidence pyramid.
- Single studies should be interpreted in the context of the entire body of scientific evidence. Science evolves, and often the results of one study may be refuted by the results of another study.

In the evidence pyramid (see Figure 1) a commentary written by a breastfeeding expert is referenced as an example of this type of literature (Renfrew, 2016). In this commentary, an expert calls for kangaroo care (extended, continuous skin-to-skin care) for infants who are small, sick, or premature (Renfrew, 2016).

## Case Reports and Case Series

Case reports are detailed reports of clinical experiences that describe the experience and outcomes of a single study participant. This study design is anecdotal and descriptive, focusing on the uniqueness of a particular case and highlighting an unusual presentation of a disease, or a novel approach to treatment (Sayre et al., 2017). Case series are descriptive and anecdotal accounts, similar to case reports, except that they describe the clinical experiences of several study participants. Case series often explore commonalities of cases, such as study participants who have the same illness, or describe experiences of patients at the same health clinic.

Case reports and case series play an important role in identifying and documenting novel and rare clinical cases, often serving as the first scientific literature on a particular subject where limited or no prior evidence exists (Sayre et al., 2017). Their role is to catalyze further research, hypotheses, or scientific dialogue. Historically, an example of a famous case series in the field of public health includes the description of five previously healthy homosexual men in Los Angeles who developed rare opportunistic infections (Gottlieb, 2006). This case series was the first official documentation of what would later be recognized as acquired immunodeficiency syndrome, known as AIDS (Gottlieb, 2006). Case reports and case series are distinct from case studies, which are qualitative, in-depth analyses and which are not included in the evidence pyramid.

Despite their usefulness in bringing forward the field of evidence-based medicine (such as spurring further research or discussion on a topic; Sayre et al., 2017), case reports and case series represent one of the lowest levels of evidence in the evidence pyramid (see the Figure 1). In the Figure 1, we provide an example of a case series that videotaped 17 mother–infant dyads to understand infant breastfeeding behaviors during skin-to-skin contact (Dani et al., 2015)

## Case-Control Studies

A case-control study is a comparison of two groups or subjects, one with an outcome of interest (cases) and one without (controls). Researchers attempt to ensure that the two groups are very similar regarding characteristics that are not specifically of interest, but which may affect the outcome of interest (such as race, age, sex, smoking status, etc.). This statistical technique is known as “matching” (Tenny et al., 2023). Then both cases and controls are studied to look for exposures that might be related to the outcome of interest. If potential exposures are associated with the outcome of interest, this may be used to build a hypothesis of the factors that may cause a particular outcome (Tenny et al., 2023). While case-control studies are very useful for finding associations between exposures and outcomes of interest, they alone cannot determine causation. This is because many exposures and outcomes may be associated with one another but are not necessarily causal. To determine whether there is a true cause-and-effect relationship between an exposure and an outcome, a prospective study design, such as a randomized controlled trial, is needed. Therefore, findings from case-control studies can inform future research and be used to design a prospective study to test the working hypothesis. Importantly, due to ethical reasons described earlier, prospective study designs are not always possible in breastfeeding and lactation research.

Case-control studies are located, therefore, in the center of the evidence pyramid. This means that they aremore robust than case reports and case series, yet less helpful than other study designs like prospective cohort studies, which demonstrate temporality and hence are more valuable in proving causality. In the evidence pyramid (see the Figure 1), we provide an example of a case-control study that examined the relationship between excessive weight loss in exclusively breastfed neonates (Jayaraj et al., 2022). The study enrolled 53 exclusively breastfed neonates (≥ 34 weeks) having weight loss of > 10% in the first 14 days of life (cases) and a group with gestational age and weight-matched neonates without significant weight loss (controls). This study found that, amongst other factors, the absence of immediate skin-to-skin contact following birth was associated with excessive neonatal weight loss (Jayaraj et al., 2022). Given our knowledge of the limitations of case-control studies, we cannot conclude that the lack of immediate skin-to-skin contact following birth alone caused excessive neonatal weight loss. A prospective study design would be needed to prove this as a causal association.

## Cohort Studies

A cohort study is designed to capture a group of participants, or cohort, and follow them over time to observe the natural progression of disease, or other outcomes of interest (e.g., exclusive breastfeeding or breastfeeding cessation; Grimes & Schulz, 2002). A cohort study can be conducted prospectively or retrospectively (Grimes & Schulz, 2002). In a prospective cohort study, the researchers start following the cohort in the present and monitor them as time moves forward. In a retrospective cohort study, researchers identify participants based on past records and follow them into the present. The purpose of this study design is to observe and measure how past or future exposures are correlated to the subjects’ health status (Grimes & Schulz, 2002). A strength of the cohort design is the ability to follow disease progression over time, especially with diseases that are slow to develop or remain latent until they are expressed (Kabeil et al., 2022). Additionally, cohort studies allow researchers to explore multiple variables and outcomes simultaneously (Kabeil et al., 2022). Cohort studies can be used to identify associations between variables and outcomes (Kabeil et al., 2022). A weakness of cohort studies is that, like many other study designs, they cannot identify causation. There is no control of variables and no intervention or control group. A cohort can be stratified based on similar exposures to variables (Kabeil et al., 2022). However, without an intervention and control group, any related variables cannot be deemed causative. This study design is therefore located in the center of the evidence pyramid. This means that its design is more robust than case-control studies, yet less helpful than other study designs, like randomized intervention trials, in proving causality.

In the evidence pyramid (see the Figure 1), we provide an example of a cohort study that examined the relationship between skin-to-skin contact following Cesarean birth and the duration of breastfeeding (Guala et al., 2017). In this study, researchers enrolled women who had a Cesarean section at a single hospital and followed them for 6 months. The participants were retrospectively divided into three groups depending on actual care in the operating room following Cesarean delivery: (1) skin-to-skin contact with the mother, (2) skin-to-skin care with the father, or (3) no skin-to-skin contact. This study showed a statistical association between skin-to-skin contact with the mother and exclusive breastfeeding rates on discharge (Guala et al., 2017).

## Clinical Trials

The key attributes of clinical trials are randomization or non-random allocation to an intervention and control group, and blinding. An intervention group is a group that is assigned some type of treatment, and a control group is a group that does not receive a treatment. The control group may be given a placebo treatment, one that is known to produce no effect, so that the participants remain unaware of the group to which they were assigned (Kandi & Vadakedath, 2023). Randomized means that participants were assigned to either an intervention or control group by chance (such as flipping a coin or pulling a number from a jar; Kandi & Vadakedath, 2023). Blinding means that either the participants, the research team, or both, do not know to which group participants are assigned (Kandi & Vadakedath, 2023).

The only research design that can definitively illuminate a cause-and-effect relationship is the randomized controlled trial (RCT). Therefore, the RCT is considered the strongest design amongst type of primary research studies scientific evidence (Mentz et al., 2016). However, these types of studies clinical trials are labor, time, and monetarily intensive, and may be subject to higher standards of ethical review than other types of studies (Mentz et al., 2016). This makes RCTs more difficult to conduct and means they may not be available to research certain phenomena.

In the evidence pyramid (see the Figure 1), we provide an example of a randomized controlled trial (RCT) that assigned infants to skin-to-skin contact directly following birth, compared to conventional care, to test the effect of skin-to-skin contact on breastfeeding outcomes (Thukral et al., 2012). Given the nature of the intervention, the researchers and health providers could not be blinded to the study groups; however, the team responsible for measuring breastfeeding behavior was blinded to the allocation. The trial demonstrated that early skin-to-skin contact did not improve breastfeeding behavior at discharge but significantly improved the exclusive breastfeeding rates of term neonates at 48 hours and 6 weeks postpartum (Thukral et al., 2012).

## Systematic Reviews

Systematic reviews are a type of secondard research, located at the top of the evidence pyramid because they not only systematically search for literature on a scientific question, but also rate the quality of included studies and summarize them.

Within a systematic review, a meta-analysis is a statistical approach that uses the findings of multiple research studies that are included in a systematic review to quantitatively synthesize and weigh them based on the quality of the primary studies. While it sounds similar, a *meta-synthesis* is a qualitative research method used in secondary research that synthesizes qualitative insights, and is hence very different than a *meta-analysis*. Some systematic reviews may use both meta-analysis and meta-synthesis to summarize findings from primary research studies.

In the evidence pyramid (see the Figure 1), we use an example of a systematic review and meta-analysis (SRMA) that examined early skin-to-skin contact across a total of nine clinical trials (Karimi et al., 2019), including the study we highlighted earlier as an example of an RCT (Thukral et al., 2012). Given that skin-to-skin contact and breastfeeding are directly observable, the researchers and health providers from many of the included trials could not be blinded to the allocation groups or the study outcome measure (breastfeeding). The SRMA demonstrated that skin-to-skin contact after birth increased the success rate and duration of the first breastfeeding (Karimi et al., 2019).

## Clinical Guidelines

Clinical Guidelines, or practice papers, are critical tools for the practicing professional. A clinical guidelines paper encompasses a comprehensive review of available scientific literature and then translates that into professional best practices. These guidelines are useful for setting institutional protocols and maintaining standards of care. Since they are written based on the best available scientific evidence, they are a convenient and accurate way to synthesize what can sometimes be a very large pool of research. Clinical guidelines also help to streamline care, make it more efficient and effective, and support clinical judgement (Youngblut & Brooten, 2001).

Many international or national professional organizations release clinical practice guidelines or practice papers. Some that may be relevant in the field of lactation can be found through organizations like the Academy of Breastfeeding Medicine, American Academy of Nursing, the Academy of Nutrition and Dietetics, the American Academy of Pediatrics, and the World Health Organization. All of these organizations release guidance on human lactation, breastfeeding, and maternal and child health.

In the evidence pyramid (see the Figure 1) we use the example of a practice guideline for skin-to-skin contact after birth, which was developed using the World Health Organization guideline development process (Brimdyr et al., 2023). Based on a systematic review and expert consultation process, the authors concluded that uninterrupted skin-to-skin contact should be the standard of care for mother–infant dyads after all modes of birth (Brimdyr et al., 2023).

## Conclusion

There are various research study designs, and these designs can be characterized according to whether they are observational or experimental, prospective or retrospective, and blinded or unblinded. All these characteristics of study designs can be used to determine the rigor of studies and their ability to prove causality.

Primary studies collect original data firsthand, whereas secondary studies, like systematic reviews and meta-analyses, summarize primary studies while assessing their scientific rigor. There can be poorly conducted scientific studies regardless of the study design. This paper provides an orientation on the concept of study designs and the evidence pyramid as an important conceptual framework that ranks study designs by their methodological rigor and susceptibility to bias. Clinical practitioners will benefit from understanding that there are a variety of potential research study designs, with both advantages and shortcomings of each type of study design. This knowledge helps to put study results into perspective and interpret results in the context of the entire body of scientific evidence, with the caveat that science evolves, and often the results of one study may be refuted by another study. Based on this knowledge, clinical practitioners can be better equipped to carefully read and assess research findings and apply them discerningly in clinical practice.

Practitioners should be aware that no single study, even a well-conducted randomized controlled trial (RCT), should be taken as definitive evidence of causality. When faced with evidence of a single RCT, practitioners should ask themselves if findings have been replicated, are biologically plausible, and whether the findings align with broader evidence. Clinicians must consider the overall strength, volume, and consistency of evidence across multiple sources, contexts, and study designs.

Finally, trust in clinical guidelines should also be informed by information on who developed them, how they were developed, and what types of studies and evidence are referenced. Solid clinical decision-making weighs the potential benefits against the potential harms of inaction, draws on evidence from peer-reviewed literature, clinical experience, informed patient consent, and other contextual factors. This ensures that evidence is not merely read, but assessed and interpreted, allowing practitioners to apply evidence responsibly while acknowledging inherent limitations.
